# High Efficacy of Oral Tetracyclines in Prosthetic Joint Infection Treated With Debridement, Antibiotics, and Implant Retention (DAIR) or Resection Arthroplasty With Destination Spacer Placement

**DOI:** 10.7759/cureus.59599

**Published:** 2024-05-03

**Authors:** Timothy L Jang, Angela Hewlett, Nicolas W Cortes-Penfield

**Affiliations:** 1 Division of Infectious Diseases, University of Nebraska Medical Center, Omaha, USA

**Keywords:** tetracyclines, destination spacer, dair, periprosthetic joint infection, prosthetic joint infection

## Abstract

Prosthetic joint infections are often managed with debridement and implant retention (DAIR) or resection arthroplasty with destination spacer placement. Both surgical approaches require long courses of postoperative antibiotics, for which tetracycline antibiotics have not been well-studied. In this retrospective case series, we included patients at our institution treated for staphylococcal prosthetic joint infection managed with DAIR or destination spacer placement who were switched from IV antibiotics to oral tetracycline within 12 weeks of surgery. Our primary outcome of interest was treatment failure within one year of initial surgery. Among the patients in our series, 88.2% (n = 15) of patients who underwent DAIR and 100% (n = 7) of patients who underwent resection arthroplasty with destination spacer remained event-free for one year. These results demonstrated that the use of oral tetracyclines as long-term therapy in the treatment of these infections was effective and well-tolerated.

## Introduction

Prosthetic joint infection (PJI) is a devastating complication of arthroplasty, requiring a combination of surgery and prolonged antibiotic therapy and carrying a high burden of morbidity (e.g., pain and loss of mobility) as well as healthcare costs.

Surgical approaches to PJI include debridement and implant retention (DAIR), 1-stage exchange arthroplasty, and two-stage exchange arthroplasty [1]. In the case of DAIR, a higher burden of residual bacteria and biofilm generally indicates a longer, and in some cases indefinite course of postoperative antibiotics. In the DATIPO trial, antimicrobial treatment for 12 weeks rather than six weeks yielded a higher rate of clinical cure in patients with PJI, with this difference being driven primarily by patients who underwent DAIR [2]. Two-stage exchange is the traditionally preferred surgical management strategy in the United States [3], but historically resulted in significant and prolonged functional impairment when static cement spacers were utilized. Fortunately, modern articulating spacers can provide full weightbearing and ambulation for a prolonged duration of time and are now sometimes being retained indefinitely as “destination spacers.”

Articulating spacers such as the PROSTALAC system (prosthesis of antibiotic-loaded acrylic cement, DePuy Synthes, Warsaw, IN, USA) or CUMARS (custom-made articulating spacers) typically combine a metal femoral stem with antimicrobial cement and a polyethylene liner [4]. While the cement component offers the advantage of releasing antibiotics directly into the joint space, a destination articulating spacer is still a large prosthetic device placed and retained in an infected site, potentially necessitating prolonged antibiotic therapy. There are few published studies describing the efficacy of destination spacers, and no guidelines specifically recommending for or against chronic antibiotic suppression after destination spacer placement. In one series of 62 PJI cases, there was no significant difference in the rate of reinfection between patients who underwent resection arthroplasty with destination spacer placement vs patients who underwent two-stage revision, although the cohort who received destination spacers did have a higher rate of noninfectious complications [5]. In another study of 51 patients who received destination spacers, chronic antibiotic suppression was not associated with fewer reinfections [6].

Existing guidelines and data recommend several well-studied antibiotic regimens for PJIs, such as IV beta-lactams or oral fluoroquinolones, often in combination with rifampin [1,7]. However, the combination of fluoroquinolones plus rifampin has the disadvantage of significant adverse effects and drug interactions. On the other hand, the tetracycline antibiotics (doxycycline, minocycline) are less-studied for the primary treatment of PJIs but offer advantages such as ease of administration/dosing, activity against commonly implicated pathogens (including staphylococci), and comparatively favorable side effect profile. Early switch to oral antibiotics has been shown to be highly effective in the management of bone and joint infections, including PJI, in multiple randomized controlled trials [8-10]. The purpose of this study was to observe the therapeutic efficacy of oral tetracyclines for staphylococcal PJI following either DAIR or resection arthroplasty with destination spacer.

## Materials and methods

We conducted a retrospective case series analysis of patients treated for staphylococcal PJI with doxycycline and minocycline at the University of Nebraska Medical Center (UNMC). Records were obtained from the outpatient parenteral antimicrobial therapy (OPAT) database, which included all patients who continued IV antimicrobial therapy for PJI upon discharge. Patients were included if they were 19 or older, underwent either DAIR or resection arthroplasty with destination spacer placement for hip, knee, or shoulder PJI between March 1, 2019 and April 1, 2021, had intraoperative cultures that grew methicillin-resistant *Staphylococcus aureus* (MRSA), methicillin-susceptible *Staphylococcus aureus* (MSSA), or coagulase-negative Staphylococci (CoNS), and switched from IV antibiotic therapy to oral tetracyclines within 12 weeks of surgery. Patients with polymicrobial infections or who received a planned second-stage revision were excluded.

The primary outcome was treatment failure one year from the date of the initial surgery for PJI, defined as reoperation for infection or PJI-related death. Data were also collected on which patients were continued on antibiotics for chronic suppression (defined as receiving antibiotics through one-year follow-up), but initiation of chronic suppression was not considered to represent treatment failure. This study was registered and approved by the UNMC institutional review board.

## Results

Of 96 patients with monomicrobial staphylococcal PJI recorded in the UNMC OPAT database during the study period, 24 met all inclusion criteria. The primary reasons for exclusion were management with two-stage revision (53.1%, n = 51) or switch to an oral antibiotic other than a tetracycline (12.5%, n = 12). Of the 24 patients included, 17 were treated with DAIR and seven were treated with resection arthroplasty with destination spacer placement. The anatomic distribution of sites included 18 knees, four hips, and two shoulders (Table 1).

**Table 1:** List of patient cases Data are shown for all patients who met the inclusion criteria (n = 24). CoNS: Coagulase-negative Staphylococci, DAIR: Debridement, antibiotics, and implant retention, MRSA: Methicillin-resistant Staphylococcus aureus

Age	Gender	Type of Surgery	Location	Pathogen Isolated	Initial IV Antimicrobials	Rifamycin?	Time until transition to doxycycline	Continued on suppressive therapy?	Reoperation for reinfection within 1 year?
82	F	Destination Spacer	Knee	CoNS	Cefazolin	Rifampin	6 weeks	No	No
55	M	Destination Spacer	Shoulder	MRSA	Vancomycin	No	5 weeks	No	No
79	F	Destination Spacer	Knee	MRSA	Vancomycin	No	6 weeks	No	No
66	M	Destination Spacer	Knee	CoNS	Vancomycin	No	8 weeks	Yes	No
68	M	Destination Spacer	Knee	MSSA	Oxacillin	Rifampin	5 weeks	Yes	No
87	M	Destination Spacer	Hip	CoNS	Cefazolin	No	6 weeks	Yes	No
86	M	Destination Spacer	Hip	MRSA	Vancomycin	No	6 weeks	No	No
79	F	DAIR	Shoulder	CoNS	Oxacillin	Rifampin	2 weeks	No	No
67	M	DAIR	Knee	CoNS	Vancomycin	No	3 weeks	No	No
69	M	DAIR	Hip	CoNS	N/A	Rifabutin	0 weeks	No	Yes
75	M	DAIR	Knee	CoNS	Daptomycin	No	4 weeks	Yes	No
60	F	DAIR	Knee	MSSA	Cefazolin	Rifampin	8 weeks	No	No
68	M	DAIR	Knee	MSSA	Cefazolin	Rifampin	6 weeks	Yes	No
85	F	DAIR	Knee	CoNS	Vancomycin	Rifampin	6 weeks	Yes	No
84	F	DAIR	Knee	CoNS	Vancomycin	Rifampin	5 weeks	Yes	No
64	M	DAIR	Knee	CoNS	Vancomycin	No	6 weeks	Yes	No
80	M	DAIR	Knee	CoNS	Cefazolin	No	6 weeks	Yes	No
61	F	DAIR	Hip	CoNS	Vancomycin	No	6 weeks	No	No
85	F	DAIR	Knee	CoNS	Vancomycin	No	9 weeks	Yes	No
87	M	DAIR	Knee	MRSA	Vancomycin	No	10 weeks	Yes	No
69	F	DAIR	Knee	MRSA	Vancomycin	No	6 weeks	Yes	No
76	M	DAIR	Knee	MRSA	Daptomycin	Rifampin	8 weeks	No	No
67	M	DAIR	Knee	MSSA	Cefazolin	No	8 weeks	No	Yes
63	M	DAIR	Knee	MSSA	Cefazolin	No	7 weeks	Yes	No

All patients except for one were initially treated with IV antibiotics, with a median duration of 42 days (interquartile range of 17.5 days) prior to switch to an oral tetracycline (doxycycline in all cases); the last patient was given oral doxycycline by his surgeon at discharge after receiving DAIR for what had been presumed to be delayed wound healing due to a postoperative hematoma, and then was continued on doxycycline with the addition of rifabutin once operative cultures yielded CoNS. IV antibiotics consisted of vancomycin or daptomycin for MRSA and methicillin-resistant CoNS, and cefazolin or oxacillin for MSSA and methicillin-sensitive CoNS. Nine patients (two of the seven destination spacers, and seven of the 17 DAIRs) received an initial course of PO rifampin (or in one case, rifabutin). All patients received at least six weeks of antibiotics.

Most patients (three of seven who underwent resection arthroplasty with destination spacer placement, and 10 of 17 who underwent DAIR) were eventually continued on suppressive antibiotics. Suppressive therapy consisted of continuing doxycycline through their one-year follow-up. Patients not on suppressive therapy at one year received a median of 11.4 total weeks of antibiotics (IV + oral), although we note that the exact duration was unable to be obtained for four of the 11 patients who did not receive suppression and were therefore unable to be included in the median. One patient experienced a rash possibly related to doxycycline, which was discontinued and replaced by trimethoprim-sulfamethoxazole to complete a total of 12 weeks of antibiotics (no chronic suppression was given in this case).

Our results showed that 88.2% (n = 15) of patients who underwent DAIR and 100% (n = 7) of patients who underwent resection arthroplasty with destination spacer remained event-free at one year (Table 2). The two patients who failed therapy both underwent DAIR and developed recurrent infection requiring additional surgery. One was the patient who had been placed on rifabutin and doxycycline immediately after surgery (treated for 12 weeks in total), and the other had been treated with eight weeks of IV antibiotics followed by 12 weeks of doxycycline for MSSA, without an adjunctive rifamycin. Neither patient had been placed on long-term suppression, though both had risk factors for treatment failure (e.g., inflammatory arthritis, infection of a revision vs primary arthroplasty). In both cases, failure occurred within 12 weeks of stopping antibiotic therapy.

**Table 2:** Patients remaining event-free at one year Data are in number event-free at one year (%). DAIR: Debridement, antibiotics, and implant retention

	DAIR	Destination Spacer
Total	15/17 (88.2%)	7/7 (100%)
Received Rifamycin		
Yes	6/7 (85.7%)	2/2 (100%)
No	9/10 (90%)	5/5 (100%)
Received Suppressive Therapy		
Yes	10/10 (100%)	3/3 (100%)
No	5/7 (71.4%)	4/4 (100%)

## Discussion

We found a high rate of treatment success with a switch to oral tetracyclines in patients with staphylococcal PJI managed with DAIR or resection arthroplasty with destination spacer placement. The success rate in our cohort compares favorably with previously reported failure rates for DAIR using alternative regimens. It suggests that oral tetracyclines may be suitable choices for oral switch in patients with staphylococcal PJI [11,12].

Adjunctive rifampin was used infrequently in this cohort, likely for two reasons: the preference of our local surgical colleagues for direct-acting anticoagulants for postoperative VTE prophylaxis, and concern about rifampin induction of doxycycline metabolism-producing subtherapeutic doxycycline levels. Despite this, treatment success was high. Use of an adjunctive rifamycin need not preclude a switch to an oral tetracycline, because rifampin does not appear to interact with minocycline and minocycline-rifampin has been reported to achieve a rate of cure similar to other non-quinolone rifampin combinations [13]. In addition, emerging in vitro, animal models, and clinical data suggest rifabutin, which avoids most relevant rifampin drug-drug interactions, might be a suitable alternative in staphylococcal biofilm infections [14-16].

The primary limitations of this study are 1) small sample size, 2) use of the OPAT database to identify patients, leading to the omission of most patients switched to oral antibiotics prior to discharge, and 3) a six-week median duration of IV therapy, which introduces immortal time bias (i.e., the majority of the cohort was at lower-than-real-world risk of treatment failure during follow-up because they had received several weeks of IV therapy before switch to an oral tetracycline, meaning early treatment failures were de facto excluded). Moreover, most patients (54%, n = 13) received suppressive antibiotic therapy, and both treatment failures occurred in the patients not given suppression; whether tetracycline therapy would be adequate without suppression is more uncertain.

## Conclusions

Our pilot data suggests that switching from IV antimicrobials to oral tetracyclines can be a safe and effective antibiotic option for the long-term therapy of staphylococcal PJI managed with DAIR or resection arthroplasty with destination spacer placement. These results were achieved without adjunctive rifampin in the majority of cases. A larger retrospective analysis or randomized controlled trial, ideally including patients who switched to oral tetracyclines earlier in the treatment course, would increase the certainty of these findings.
